# Extreme plasticity in reproductive biology of an oviparous lizard

**DOI:** 10.1002/ece3.4247

**Published:** 2018-06-11

**Authors:** Mats Olsson, Lisa Loeb, Willow Lindsay, Erik Wapstra, Luisa Fitzpatrick, Richard Shine

**Affiliations:** ^1^ Division of Animal Ecology Department of Zoology University of Gothenburg Gothenburg Sweden; ^2^ School of Biological Sciences University of Wollongong Wollongong NSW Australia; ^3^ School of Biological Sciences University of Tasmania Hobart Tasmania Australia; ^4^ School of Life and Environmental Sciences University of Sydney Sydney NSW Australia

**Keywords:** developmental plasticity, incubation, Lacertidae, reproductive mode

## Abstract

Most oviparous squamate reptiles lay their eggs when embryos have completed less than one‐third of development, with the remaining two‐thirds spent in an external nest. Even when females facultatively retain eggs in dry or cold conditions, such retention generally causes only a minor (<10%) decrease in subsequent incubation periods. In contrast, we found that female sand lizards (*Lacerta agilis*) from an experimentally founded field population (established ca. 20 years ago on the southwest coast of Sweden) exhibited wide variation in incubation periods even when the eggs were kept at standard (25°C) conditions. Females that retained eggs in utero for longer based on the delay between capture and oviposition produced eggs that hatched sooner. In the extreme case, eggs hatched after only 55% of the “normal” incubation period. Although the proximate mechanisms underlying this flexibility remain unclear, our results from this first full field season at the new study site show that females within a single cold‐climate population of lizards can span a substantial proportion of the continuum from “normal” oviparity to viviparity.

## 1 INTRODUCTION

Reproductive modes in squamate reptiles (lizards and snakes) traditionally are divided into oviparity (egg‐laying) and viviparity (live‐bearing) (Blackburn, 1993). Most oviparous species lay their eggs after the embryos have completed around one‐third of total development time (stages 25–33 in Dufaure and Hubert (1961) system), whereas viviparous species produce fully developed offspring (stage 40). Intermediates between these two endpoints are rare (Andrews & Mathies, 2000; Blackburn, 1995; DeMarco, 1993; Shine, 1983). Thus, although oviparity and viviparity lie on a continuum, most species cluster at the ends of that continuum, completing either 20%–30% or 95%–100% of embryonic development in utero (Blackburn, 1995).

Prolonged uterine retention of eggs in squamates is linked to ambient temperatures, with more prolonged uterine retention of eggs in cooler climates (e.g., Huey, 1977; Mathies & Andrews, 1996; Neill, 1964; Smith & Shine, 1997). Such geographic shifts in incubation period (i.e., postoviposition to hatching) could be either facultative or hard‐wired. In one of the earliest discussions of viviparity in reptiles, Weekes (1933) suggested that viviparity arises as a direct (phenotypically plastic) response to low temperatures. Subsequent research rejected that view, but recent experimental studies have confirmed that exposure to dry conditions or low temperatures can induce females to retain their eggs in utero for longer than usual (Stamps, 1976; Telemeco, Radder, Baird, & Shine, 2010; Warner & Andrews, 2003). Nonetheless, such prolongation typically has little effect on subsequent incubation periods (Mathies & Andrews, 1999; Rodriguez‐Diaz & Brana, 2010; Shanbhag, Saidapur, & Radder, 2003; Telemeco et al., 2010; Warner & Andrews, 2003). In *Sceloporus aeneus* and *S. scalaris*, however, oviductally retained embryos continue to develop in utero, reducing the subsequent incubation period (Andrews & Mathies, 2000). At the extreme, *S. scalaris* eggs hatched in <3 days after surgical removal from uteri (Andrews & Mathies, 2000; Mathies & Andrews, 1999). In *Lacerta vivipara*, Foucart, Heulin, and Lourdais (2017) estimated that fecundity‐enforced trade‐offs shifted the proportion of total developmental time spent in utero from about 31% to 38%. Because eggs increase in mass (due to water uptake) as they develop, prolonged retention of eggs in utero will increase physical burdening of the gravid female and may require a reduction in the number of offspring in order to maintain equivalent clutch volume.

**Figure 1:**
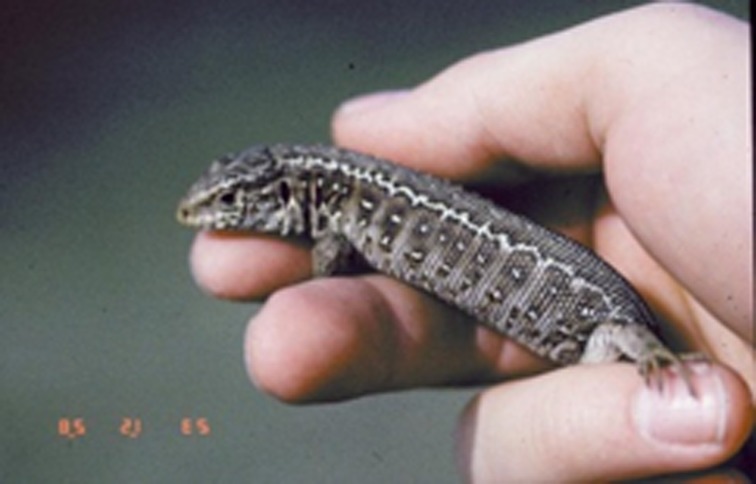
Female sand lizard (*Lacerta agilis*)

The sand lizard (*Lacerta agilis*; Figure 1) occurs across a massive geographic range in Europe, encompassing a broad range of environments (Roitberg et al., 2015). Incubation periods of eggs are briefer in cool‐climate populations than warm‐climate populations, putatively reflecting selection for rapid development in cool conditions (Roitberg et al., 2015; Rykena, 1987; While et al., 2015a, 2015b). In a population near the northern edge of the species’ range in Sweden, incubation periods are also shorter in clutches laid in cooler summers, and in clutches laid late within a season (Shine, Wapstra, & Olsson, 2017). Those correlations suggest that natural selection has favored a facultative adjustment of incubation period to match the temporal window of high soil temperatures available for incubation (i.e., before soil temperatures fall to lethally low levels at the onset of winter: Shine et al., 2017).

In the present paper, we focus on the degree to which environmental conditions stimulate shifts in incubation periods in sand lizards. Over the 10 years of our previous study on a nearby population (Olsson, Gullberg, Shine, Madsen, & Tegelström, 1996; Olsson, Gullberg, & Tegelström, 1996; Olsson & Shine, 1997a, 1997b), mean incubation periods (at a constant 25°C) averaged 39.5 days (*SE* = 0.17, *n *= 420 clutches), with only one clutch (at 28 days incubation) hatching in less than 30 days (Shine et al., 2017). In 2017, during research on a nearby (experimentally founded) population of the same species, we recorded more extreme variation in incubation periods; even at constant 25°C, some eggs hatched after little more than half the “normal” incubation period. In the current paper, we describe and interpret this plasticity.

## 2 MATERIALS AND METHODS

Sand lizards occur widely across Europe (Roitberg et al., 2015); our study sites are near the northern limit of the species’ range in southwestern Sweden. Most of our research has been at a mainland (coastal) site at Asketunnan (57°22 N, 11°59′ E), but this population has declined in recent years, and we have shifted our focus to a small island (St. Keholmen, 57°29′ N 11°56′ E) ca. 15 km to the north. The island is separated by a ca. 100 m of shallow (<1 m) water from the adjacent mainland, but surveys over many years never revealed any reptiles on the site, despite what appeared excellent sand lizard habitat. In 1999 and 2000, sand lizards were captive‐bred and females were given ad libitum food to initiate a second clutch and thus double female reproductive output. Males and females from southern Sweden were outbred between local populations to counter the effects of inbreeding at our main study site (Asketunnan; Olsson, Gullberg, & Tegelström, 1996; Olsson, Gullberg, Shine, et al., 1996). The 454 offspring (all <1 month of age) from 75 females that had copulated with 51 first males and 35 second males, in single or double (sperm competition) matings, were released on the site to found a new population, with more genetic variation than Asketunnan. Adults were then released back to their site and place of capture. Thus, the colonizing lizards had a diverse genetic background compared to Asketunnan, with parents also collected from areas further south in Sweden where the climate is warmer and the species is more continuously distributed (Berglind, Gullberg, & Olsson, 2016). We returned to the island site in 2017 to capture lizards and obtain eggs. Elsewhere, we report on the increased hatching success and reduced risk of malformations associated with this increased genetic variation (Loeb et al., submitted).

In the wild, female sand lizards in this region copulate for about 2.5 min, are mate guarded for up to several weeks, and thereafter produce only a single clutch of eggs per year. The duration of the egg‐laying period depends on weather but is approximately 6 weeks long (Ljungström, Stjernstedt, Wapstra, & Olsson, 2016; Ljungström, Wapstra, & Olsson, 2015; Olsson & Shine, 1997a, 1997b). We visited the island on every day with suitably warm weather during the lizards’ activity season, and females with oviductal eggs (evident from palpation and the animal's distended body shape) were returned to our laboratory. They were kept individually in cages (500 × 400 × 350 mm) with a sand substrate and a flat rock over moist soil for egg laying. Ambient temperature was 18°C, but a 40 W spotlight at one end of each cage enabled females to attain body temperatures of up to 40°C if they chose to do so. Newly laid eggs were immediately removed and incubated (one clutch per container) in moist vermiculite (ca. 1:8 water to vermiculite by volume) at constant 25°C, a temperature that minimizes developmental abnormalities (Zakharov, 1989).

Statistical analyses using JMP 11 were based on clutch means rather than individual eggs as the unit of replication (all eggs within a clutch invariably hatched on the same day). For comparison, we combined the 2017 data from the island population with data gathered earlier (1998–2007) on the Asketunnan population (see Shine et al., 2017). We compared mean incubation periods of the eggs of mainland versus island lizards using one‐factor ANOVA; data were ln‐transformed to achieve normality and variance homogeneity. To explore possible interactive effects of location and time in captivity prior to oviposition on incubation periods, we included the latter variable as a covariate in homogeneity of slopes tests (again, with incubation period ln‐transformed). We compared frequency distributions of incubation durations between the two sites using the nonparametric Kolmogorov–Smirnov test. We used Pearson product–moment correlation analyses to quantify relationships among traits.

## 3 RESULTS

**Figure 2:**
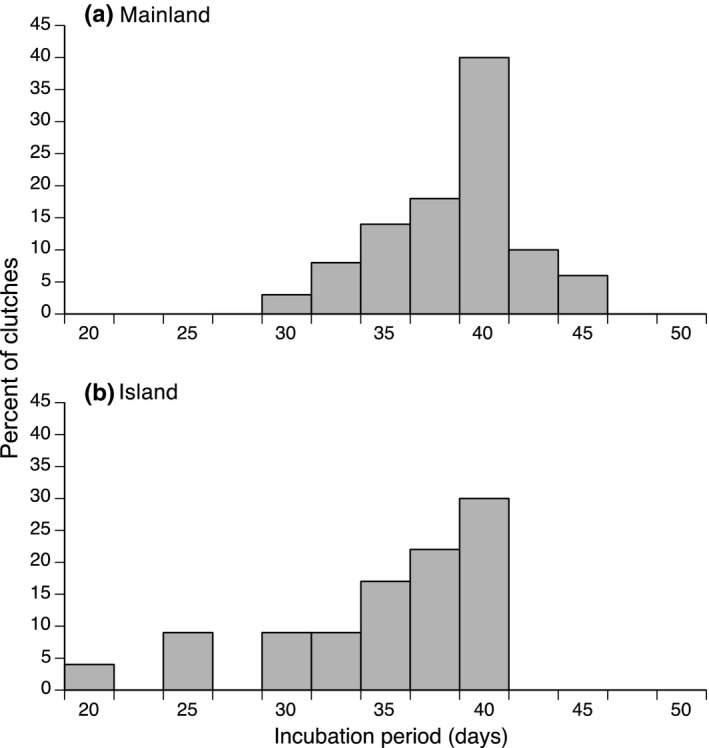
Incubation periods of the eggs of sand lizards (*Lacerta agilis*) from two populations in southern Sweden. When incubated at a constant 25°C, the eggs of lizards from a mainland population (a) hatched later than eggs from an island population (b)

The eggs of island lizards hatched sooner than did those of mainland conspecifics (means of 36.05, *SE* = 0.76 days vs 39.53, *SE* = 0.18 days; ANOVA on ln‐transformed data *F*_1, 442_ = 23.66, *p* < 0.0001). That relatively small (3.5 day) difference in mean incubation periods masked a major difference in frequency distributions of incubation periods between the two populations (Figure 2). Whereas the frequency distribution of incubation periods for mainland lizards was normal, the one for island lizards was strongly left‐skewed (Figure 2). As a result, several clutches of island lizards hatched after a briefer incubation period than was ever recorded for the much larger sample of mainland clutches (Figure 2). Of 23 clutches from island lizards, 13% hatched in <30 days, and the minimum incubation period (20 days) was 55% of the mean value, whereas for 420 clutches of mainland lizards the shortest incubation period (28 days) was greater than 70% of the mean. A Kolmogorov–Smirnov test confirmed that the two frequency distributions differed significantly (*n* = 443 clutches, KS = 0.062, KSa = 1.312, *p* < 0.035).

**Figure 3:**
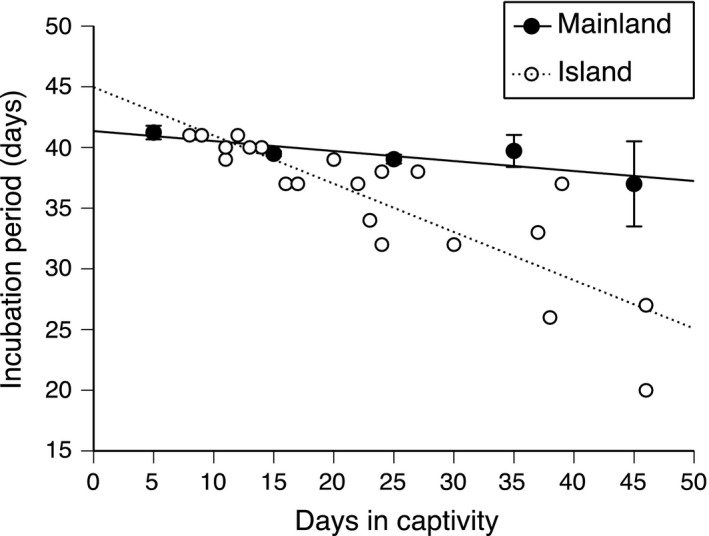
Incubation periods of the eggs of sand lizards (*Lacerta agilis*) from two populations in southern Sweden, as a function of the interval between capture of a gravid female and her oviposition in the laboratory. Data for 420 clutches of mainland females are taken from Shine et al. (2017; see that study for more details); to avoid cluttering the graph, we show mean values and *SE*s for each 5‐day interval of durations of maternal maintenance in captivity prior to oviposition. In contrast, data for island females show mean values for 23 clutches and thus do not have associated error terms (all eggs within a clutch hatched on the same day)

Among the island clutches, incubation duration was not significantly correlated with mean offspring mass (*r* ^2^ = 0.003, *n* = 23, *p* = 0.80), but was linked to the length of time females were held in captivity between their capture and oviposition. As in the Asketunnan population (Shine et al., 2017), incubation periods of eggs laid by the island lizards hatched after briefer incubation when a female had been retained in captivity for a longer period and delayed oviposition (*r* ^2^ = 0.72, *n* = 23, *p* < 0.0001). Island females delayed laying their eggs until they had been in captivity for an average of 22.40 days (*SE* = 1.123) compared to 15.33 days (*SE* = 0.25) for mainland lizards (*F*_1,569_ = 32.08, *p* < 0.0001). In addition, incubation periods of the eggs of island females exhibited a more rapid decline with increasing durations of time that the mother was held in captivity (Figure 2; Homogeneity of slopes test with incubation period as dependent variable, location as factor, and days in captivity as covariate: interaction location × days in captivity *F*_1, 438_ = 27.48, *p* < 0.0001). Thus, incubation periods were shorter in island clutches than mainland clutches because (a) females delayed laying their eggs for longer periods after capture, and (b) a given period of maternal maintenance in captivity reduced subsequent incubation periods more rapidly in island eggs than in mainland eggs (Figure 3).

## 4 DISCUSSION

We show that reproductive output of females from an island population of sand lizards in 2017 was similar to that we have documented in a nearby mainland population in earlier years (Shine et al., 2017); however, females that delayed ovipositing (and hence were held in captivity for a longer period before laying) produced eggs with briefer subsequent incubation periods. The magnitude of this effect differed substantially between the two populations. Females from the island population delayed laying for longer and produced eggs with brief (and strongly left‐skewed) subsequent incubation periods (Figure 2). Even at similar durations of captivity prior to oviposition, island clutches hatched sooner than mainland clutches (Figure 3).

Although these interpopulation differences are clear, the proximate mechanisms that underpin them are difficult to tease apart. The plausible candidates are that short incubation periods in island eggs are due to (a) maternal genes (local adaptation or pre‐adaptation); (b) island environments (e.g., low ambient temperatures?); or (c) gene × environment interactions (i.e., the brief incubation reflects a norm of reaction of island lizards triggered by the ambient conditions of the island environment). Below, we consider these possibilities.

### 4.1 Maternal genes

We doubt that the brief and variable incubation periods of the island lizards reflect their genetic heritage (admixture of warm‐climate populations), because warmer‐climate sand lizards produce eggs with longer not shorter incubation periods (Roitberg et al., 2015). More plausibly, selection over the almost 20 years (approximately four generations) of island life may have favored a shift toward prolonged uterine retention of eggs and thus an abbreviated subsequent incubation. This latter process has been documented in lacertid lizards (*Podarcis muralis*) translocated to cooler climates (Italy and France to the UK), over a similar time frame (20–80 years; While et al., 2015a). However, the within‐population variance in incubation periods was lower in that study (minimum incubation duration >87% of mean incubation period, across all populations at both temperatures tested: While et al., 2015b) than in the Swedish sand lizards (minimum incubation duration 55% of mean incubation period for the island lizards, 71% for Asketunnan lizards).

### 4.2 Island environments

We have no data on soil temperatures on our Swedish study sites, but they are probably cooler than most other areas that support sand lizards (Shine et al., 2017). Thus, females may have responded to low ambient temperatures by facultatively accelerating embryonic development via prolonged uterine retention of eggs (as has been documented in other lizards: Telemeco et al., 2010). The strong correlation between duration of time in captivity prior to oviposition versus subsequent incubation period (Figure 3) suggests that shorter incubation results from more advanced embryogenesis at laying. Data on stages of embryonic development at laying would be needed to test that hypothesis, and we have no such data.

### 4.3 Gene × environment interaction

The brief and highly variable incubation periods of island lizards may reflect the impact of a cool ambient environment on norms of reaction of lizards from a relatively warm‐climate lineage. Squamates with little opportunity to maintain high body temperatures tend to select warmer‐than‐usual temperatures when given that opportunity. For example, an experimentally imposed reduction in basking opportunity induced lizards to select higher temperatures during the brief period when such opportunities were available (Shine, 2006) and basking behavior was more prolonged in snakes that had previously been maintained under cool conditions than in their siblings that had been maintained at higher temperatures (Aubret & Shine, 2010). By analogy, island lizards (from warm‐climate lineages) may have exploited the basking opportunity offered in captivity, to a greater degree than would local (cold‐adapted) Asketunnan conspecifics. Such a difference in maternal thermal regimes prior to oviposition could explain why an additional day between capture and oviposition accelerated embryogenesis more in island lizards than in their mainland counterparts (Figure 3). Data on basking behavior and body temperatures could enable a test of this hypothesis.

Regardless of the proximate mechanisms generating variation in incubation periods of eggs from our island study population, the magnitude of that variation is interesting in its own right. In combination with previous studies (notably, those of Mathies & Andrews, 1996), our results suggest that variation (likely facultative) in the duration of retention of developing eggs in utero can generate substantial corresponding variation in incubation periods among clutches within a population. In the case of the island sand lizards, that variation encompasses about half of the disparity between “normal” oviparity versus viviparity. Highly abbreviated incubation periods are rare among oviparous squamates (Blackburn, 1995) and have been reported primarily in populations from areas close to the lower thermal limits in which oviparity is possible (Huey, 1977; Mathies & Andrews, 1996; Smith & Shine, 1997). Our data fit that generalization. To our knowledge, however, our data are the first to show such wide variation in incubation periods of eggs from females within a single population.

## CONFLICT OF INTERESTS

The authors declare no conflict of interests.

## AUTHORS CONTRIBUTIONS

M.O. and E.W. conceived the field study. All authors performed fieldwork. R. S. wrote the original MS, and all authors commented on an earlier draft of the manuscript.

## DATA ACCESSIBILITY

Data will become available on Dryad Digital Repository upon manuscript acceptance for publication.
